# Interaction effects of pen environment and sex on behavior, skin lesions and physiology of Windsnyer pigs

**DOI:** 10.5713/ajas.17.0417

**Published:** 2019-02-20

**Authors:** Mbusiseni Vusumuzi Mkwanazi, Arnold Tapera Kanengoni, Michael Chimonyo

**Affiliations:** 1 Animal and Poultry Science, School of Agricultural, Earth and Environmental Sciences, University of KwaZulu-Natal, P Bag X01 Scottsville 3209, Pietermaritzburg 3209, South Africa; 2 Veterinary Services and Research Development, Joburg Zoo, Private Bag X 13, Parkview 2122, South Africa; 3 College of Agriculture and Environmental Sciences, University of South Africa, Florida Campus, Florida 1709, South Africa

**Keywords:** Aggressive Behaviour, Animal Welfare, Exploratory Behaviour, Heart Rate, Pen Fixtures, Respiration Rate

## Abstract

**Objective:**

The study was carried to determine the interaction effects of pen enrichment and sex on behavioral activities, skin lesions and physiology of Windsnyer pigs.

**Methods:**

Forty-eight growing Windsnyer pigs of both sex, with an average initial body weight of 21.6 (±9.01) kg were used. Four pigs were randomly assigned to either enriched or barren pens at a stocking density of 0.35 m^2^/pig. Enriched pens contained 2 L bottles filled with stones and suspended at head level on ropes stretching across the pens. In addition, two plastic balls (90 mm in diameter) and 500 mL bottles (235 mm long) were placed on the floor of each enriched pen.

**Results:**

Pigs in barren environments had higher heart rates (p<0.001) than those in enriched pens. There was an interaction of pen environment and sex on rectal temperature (p<0.001). Females in enriched pens had higher rectal temperatures (p<0.05) than females in barren pens. There was no interaction of pen environment and sex on time spent eating and drinking (p>0.05). Time spent bullying was influenced (p<0.05) by pen environment and sex. Female pigs in barren environment spent more time on bullying than females in enriched pens. There was an interaction of pen environment and sex on time spent lying down and walking (p<0.05). Female pigs in enriched pens spent more time lying down than females in barren pens. Males in barren pens spent more time walking than males in enriched pens while no effect of pen environment was observed in females. There was an interaction of pen environment and sex on the number of skin lesions in the head, neck and shoulder region and other parts of the body (p<0.05).

**Conclusion:**

It was concluded that pen enrichment reduced the number of skin lesions and anti-social behaviors, especially for female pigs. There is a need, therefore of housing indigenous pigs under confinement.

## INTRODUCTION

Pig production is generally increasing in the Sub Saharan Africa because of growing human population. Local genotypes, such as Windsnyer pigs that are adapted to local conditions and thrive on fibrous feeds can be of much value with regard to feeding the growing population. The Windsnyer pig is short framed, with long nose and razor back, and has an average mature weight of 100 kg [1]. Windsnyer pigs are heat tolerant and are likely to survive extreme temperatures which are largely faced by farmers in Southern Africa. The merits of this breed are, however, not fully exploited to improve food security. To conserve the genetic diversity of the Windsnyer population more research is needed especially on housing them under commercial conditions. The emphasis by national institutions on increasing productivity has led to the dominance of high productive imported breeds in Southern Africa [2]. In South Africa, for example, the organization which has the mandate to improve pig production exclusively deals with imported breeds such as Large White, Landrace and Duroc. The preference of imported breeds has contributed to the lack of improvement of slow growing Windsnyer pigs, thereby threatening their existence.

The welfare of growing–finishing pigs can directly influence performance variables such as average daily gain, average daily feed intake, and feed conversion ratio. Growth performance alone, however cannot accurately reflect and assess the extent to which the welfare of the pigs is met [3]. In addition to growth performance, indicators related to behavior and physiology should be considered. Information on physiological responses during exposure to pen environment is limited but could be measured through variations in heart rate (HR), respiration rate and rectal temperature [4]. More so, indicators of poor welfare such as skin lesions and bruising can assist in assessing pig welfare in relation to pen enrichment. Research has consistently reported that enriching the pen environment improves welfare and physiological response of pigs [5–6]. These studies, however, have only been focused on exotic breeds and did not determine possible interactions between pen environment and sex on behavior and physiological responses of indigenous pigs.

Due to differences in frame size and temperament between the Windsnyer and exotic breeds, it is likely that the extent to which pen enrichment affect their behavior differs. Most commercial farmers mix male and female pigs during fattening. The female Windsnyer pigs are, however likely to exhibit estrus before they are ready for slaughter [2]. Puberty is also likely to influence any degree of activity within a pen and could, therefore interact with any attempts to enrich pig pens. There is need to determine the extent to which pen environment can influence the behavior and physiological responses of male and female Windsnyer pigs. The objective of the study was to determine the interaction of pen environment and sex on behavioral activities, skin lesions and physiology of Windsnyer pigs. It was hypothesized that there is interaction between pen environment and sex on physiological responses and behavior of Windsnyer pigs.

## MATERIALS AND METHODS

### Ethical consideration

The experimental procedures were performed according to the ethical guidelines specified by the Certification of Authorization to Experiment on Living Animals provided by the Agricultural Research Council (ARC) - Animal Ethics Committee (Reference No: APIEC 16/016).

### Study site

The trial was carried out at the Pig Production unit of the Agricultural Research Council (ARC) Animal Production Institute at Irene, South Africa. It lies at about 25°34′0″S and 28°22′0″E and is approximately 1,526 m above sea level. The average annual temperature is 18.7°C.

### Pigs, feeding, and housing management

Forty-eight clinically healthy growing Windsnyer pigs, with a mean (±standard deviation) body weight of 21.6 (±9.01) kg were used in the study. All pigs were managed under a small scale intensive production system and were under a feeding regime that was designed to mimic conditions that are experienced in small holder farming areas. No feeding standards are available for Windsnyer pigs. The pigs were scavenging freely on forages and given pumpkins and kitchen waste. The houses had no creep area, farrowing crates or infrared lamps. The weaning age for Windsnyer pigs is approximately 8.5 to 9 kg. Prior to introduction to their experimental pen, pigs had no exposure to environmental enrichment. The pigs were then selected randomly from the pig herd and assigned four to each pen according their sexes. The pigs were left to adapt to their new environment for seven days before being monitored for 42 d. The period would also allow them to establish ranks so that the effect of enrichment and sex on behavior and physiology could be clearly defined. The pigs were penned with a space allowance of 0.39 m^2^ pens in average and were fed on a commercial standard diet (Supreme Grower, Meadow Feeds Ltd, Pretoria, South Africa) throughout the duration of the study. Feed was provided *ad libitum* from one tube feeder permanently fixed at the centre of the pens. Water was also provided *ad libitum* from low pressure nipple drinker located on the wall of the pen. The room temperature and humidity were 24.5(±1.9)°C and 62.7(±15.07)%, respectively. The housing facility was cleaned and disinfected using Virkons (Pretoria, South Africa) disinfectant before the commencement of the trial.

### Experimental design, treatments and enrichment structures

The study was conducted through a 2×2 (pen environment× sex) factorial arrangement. Pigs were blocked by sex and randomly allocated to 12 pens resulting in 4 pigs per pen. Twenty-four animals (12 intact males, 12 females) were housed in physically enriched pens and 24 animals (12 intact males, 12 females) housed in barren pens. Enriched pens contained 2 litre bottles filled with stones and suspended at head level on ropes stretching across the pens. The bottles were 350 mm long and placed 650 mm apart. Also, two plastic balls (90 mm in diameter) supplied by (PLEXX BV, Elst, Netherlands) and 500 mL bottles (235 mm long) were placed on the floor of each enriched pen. The enrichment items used were chosen based on their ease of availability and low cost. The enrichment items placed on the floor were periodically cleaned three times a week to remove feces following the pig’s activity. No enrichment of any kind was applied to barren environment pens. Experimental pigs were not subjected to tail docking during the study.

### Measurements

#### Physiological responses

To measure physiological responses pigs were confined in a 1.3×0.5×0.8 m metal cage in the same room with minimum restraint. Rectal temperature (RT), respiratory rate (RR), and HRs were measured. The cage had steel bars on its side and lockable doors on both ends. The pigs were handled three times a day for period of seven days prior the beginning of the experiment for them to get accustomed to the stockmen and measurement routine. Physiological measurements of pigs were measured every day for a period of 12 days at 0800 h.

The RT of the pigs was measured using a digital thermometer (Uniontech, Suzhou, Jiangsu, China) at the same time when body weight (BW) was measured. Temperature was measured and recorded. The thermometer was inserted in the rectum to full depth until a stable automated reading was obtained. The RR (in beats per minute) for each pig was measured on the same day when BW and RT were measured. The measurements were done visually by observing and counting the movements of the flank of each pig. The movements of the flank were observed for 15 seconds. The HR of the pigs was measured using a stethoscope. The stethoscope was placed on the artery below and slightly inside the jaw. The HR was observed for 15 seconds and multiplied by four to obtain the number of heart beats per minutes. The HR was expressed as beats per minute.

### Pig behavioral recordings

Behavioral activities of pigs in each pen were recorded continuously using a ceiling mounted video camera, Sony handycam dcr −hr 52/54 (Game, Pretoria, South Africa). The use of video cameras was to prevent disturbances from observers to the pigs whilst they are exhibiting their normal day to day activities. Pigs were identified with paint markings at the back for accurate identification during observation. Time spent on different behavioral activities was observed six times a week for a period of three weeks (21 d) for eight hours, starting from 0800 h and ending at 1600 h as this part of the day has been described as the most active time for growing pigs [7] In each pen, two focal pigs were selected, similar experimental numbers of pigs we used by Beattie et al [6] and [8] these were selected based on their body weight, pigs with a body weight between 25 and 30 kg, 7 days after the beginning of the experiment. The same focal pigs were observed throughout the trial. The behavioral activities were scored by the same person during the study period. Behavioral activities observed in the study are based on the Ethogram given in Table 1.

### Skin lesions

Skin lesions emanating through aggression and frequency of fighting were assessed on individual pigs in each pen. Recordings were done on fresh lesions which were judged subjectively by colour and the estimated age of scabbing [9,10]. Lesions on each side of the pig were added to obtain a total of each side then score based on the total number of lesions. The scoring method used followed that of Gonyou et al [9] which is; 0 = no lesions, 1 = 1 – 3 lesions, 2 = 4 – 6 lesions, 3 = more than 6 lesions.

For the purpose of assessment, the body of the pigs was divided into three zones per side (head; neck and shoulders and other parts). Each zone received a score based on the number of lesions on it. To keep the scores consistent throughout the experiment one person, assessed the skin lesions.

### Statistical analyses

For the analyses of behavioral data, PROC UNIVARIATE (SAS 2016) was used to check the data for normality. Logarithmic transformation was used to normalize data for injuries. Data for physiological responses, behavioral activities and skin lesions was analysed using PROC MIXED for repeated measurements. The effect of environmental temperature at the time of measurement was not significant, therefore it was removed from the model. The effect of day was used as a within-subject’s variable. During the analyses of HR, RT, and RR, data for each pig was used as the experimental unit. For behavioral activities and skin lesions the pen was considered as the experimental unit. The model used was:

$${\text{Y}}_{\text{ijk}}=\mu +{\text{S}}_{\text{j}}+{\text{P}}_{\text{k}}+{\text{W}}_{\text{l}}+{\left(\text{S}\times \text{P}\right)}_{\text{jk}}+{\left(\text{S}\times \text{W}\right)}_{\text{jl}}+{\left(\text{P}\times \text{W}\right)}_{\text{kl}}+{\left(\text{S}\times \text{P}\times \text{W}\right)}_{\text{jkl}}+\beta_{1}\text{BWT}+{ɛ}_{\text{ijkl}}$$

Y_ijk_, is the response variable (pig behavior observations, skin lesions, RT, HR, RR); μ is the overall mean common to all observation; S_j_ is the effect of sex (male, female); P_k_ is the effect of pen environment (enriched, barren); W_l_ is the effect of week; (S×P)_jk_ is the interaction of sex and pen environment; (S×W)_jl_ is the interaction of sex and week; (S×P×W)_jkl_ is the interaction of sex, pen environment and week, BWT is the effect of initial body weight; β_1_ BWT is the partial regression coefficient of the dependent variable on BWT and ɛ_ijkl_ is the residual error. Differences among the least square means were considered significant at p<0.05.

## RESULTS

### Physiological responses

There was a significant effect of initial body weight incorporated as covariates (p<0.05) on HR, RR, and RT. There was no interaction of pen environment and sex on HR of pigs (p>0.05). The HR was, however, influenced by pen environment (p< 0.001). Pigs in barren environment had higher HR than pigs in enriched environment (Table 2).

There was an interaction between pen environment and sex on RR (p<0.05). Female pigs in barren environment had higher RR than female pigs in enriched pens; however, male pigs had similar RR regardless of the environment. There was an interaction between pen environment and sex on RT (p< 0.001). Male pigs in barren environment had higher RT than enriched male pigs, respectively. Enriched female pigs had higher RT than females in the barren environment (Table 2).

### Behavioral observations

There was no interaction of pen environment and sex on time spent eating and drinking (p>0.05). Time spent eating and drinking was similar regardless of environment (Table 3). There was no interaction of pen environment and sex on time spent fighting (p>0.05). Pen environment, however, affected time spent fighting (p<0.001). Independent of sex, pigs spent more time fighting in barren environments (Table 3).

There was an interaction of pen environment and sex on time spent bullying (p<0.05). Female pigs in barren environments spent more time bullying than females in enriched pens while no effect of pen environment was observed in males. There was no interaction of pen environment and sex on time when the pig was actively standing (p>0.05). There was, however, an interaction of pen environment and sex on time spent lying down and walking (p<0.05). Female pigs in enriched pens spent more time lying down than females in barren pens whilst male pigs spent similar amount of time lying regardless of being in enriched or barren pens (Table 3). Environment affected time spent walking (p<0.05). Males in barren pens spent more time walking than males in enriched pens; but females regardless of the environments spent similar amount of time walking. There was an interaction of pen environment and sex on time spent mounting other pigs (p<0.05). Female pigs in barren pens spent more time mounting than females in enriched pens. The pen environment did not affect time spent mounting in male pigs (Table 3). The interaction of pen environment and sex on time-spent head rubbing was not significant (p>0.05). The proportion of time spent on exploratory behaviors was unaffected by the interaction of pen environment and sex (p>0.05).

Female pigs in enriched pens spent similar amount of time exploring the substrates as did male pigs. Time spent exploring pen fixtures was affected by pen environment (p<0.001), with males and females in barren pens spending more time exploring pen fixtures than male and female pigs in enriched pens (Table 3). There was a significant interaction between pen environment and sex on time spent performing other behaviours (rolling, pivoting, and tail biting) (p<0.05). Male pigs in barren pens spent more time on behaviours described as others, which were mainly tail biting, rolling and pivoting than females, while, enriched male, and female pigs spent similar amount of time on those behaviours.

### Skin lesions

Table 4 shows the interaction of pen environment and sex on skin lesions. There was an interaction of pen environment and sex on skin lesions found in the head region, neck and shoulder region and other parts (p<0.05). Female pigs in barren pens had more head skin lesions compared to females in enriched pens. The pen environment, however, did not affect the number of skin lesions of in the head region in male pigs. Female pigs in enriched pens had fewer lesions on the neck and shoulder region compared to females in barren pens. Pen environment did not affect the number of lesions on the neck and shoulder region in male pigs. Enriched female pigs had fewer lesions on the other parts of the body compared to female pigs in barren pens. The pen environment, however, did not result in any lesions in male pigs.

## DISCUSSION

The preference of exotic pigs has led to a decline in the population of indigenous breeds such as Windsnyer pigs. An estimated 16% of the uniquely adapted genotypes have been lost over the last century [1]. In some Southern African countries, when a few indigenous breeds are reduced by natural disasters or civil wars, it is replaced by modern commercial breeds, leading to a reduction of genetic diversity. High fiber digestibility is of importance in rural areas where resources are limiting. The HR was within the normal range for the pigs. The normal HR range is between 70 and 107 beats/min [4]. The observation that HR was influenced by pen environment was not surprising. The higher HR for male pigs in barren pens could be an indication of arousal because of physical activities amongst the pigs with no enrichment. The finding that pigs in enriched environment had lower HR could reflect being calmer and resting more because of spending more time on exploring the substrate [4].

The normal respiration rate for pigs is between 15 and 30 beats/min. Caldara et al [4] reported that respiration rate did not differ between pigs enriched with wood shavings, compact floor and coffee husks bedding. The finding that female pigs in barren pens had higher RR than females in enriched pens could indicate increased stress levels in the female pigs. The higher RT for male pigs in barren environment could suggest that they were trying to maintain a temperature gradient between core and skin temperature [11]. Stress can also affect body temperature rhythm and body temperature level. Therefore, differences in rectal temperature can also be explained by differences in circadian rhythm. However, to elucidate this concept further research still need to be done to evaluate how pen enrichment affect circadian rhythm or body temperature. There is also need of understanding the relationship between HR, RT, and RR during environmental enrichment.

The finding that there was no interaction between pen environment and sex on time spent eating and drinking was surprising because in the absence of enrichment substrates in barren environment you would expect pigs to spend more time eating and drinking. A possible explanation could be that pigs in enriched pens were restricted in their use of enrichment substrates because of the size and pen design such that when one pig is feeding, it would effectively block the movement of others. More time spent drinking by pigs in barren environment was expected because of boredom, therefore pigs could be playing with the nipple drinker.

Windsnyer pigs have a calmer temperament than the fast-growing pigs that could possibly suggest the lack of interaction of pen environment and sex on time spent fighting. The finding that pigs in barren environment spent more time fighting than pigs in enriched environment could have emanated out of frustration when queuing at the feeder or due to competition of feed within the pen. Fighting could have been reduced if more feeders were made available to reduce competition for access to feed. Fighting may also have been elicited by discomfort, irritability and annoyance in pigs [7]. The reduction of fighting with enrichment may not be a direct consequence of the enrichments but rather a secondary result associated with the decrease in harmful social behaviour that is brought by the enrichment [12]. Bullying behavior in the study was defined as one pig being dominant over the other in a fight. The observed interaction on bullying with female pigs in barren environment spending more time bullying could have reflected a dominant pig being in control of the pen environment and submissive pigs not retaliating to avoid social encounters or choosing to wait for the dominant pig to stop its activity before it does what it desires to do.

The unexpected observation that there was no interaction between pen environment and sex on time spent actively standing is surprising as Windsnyer pigs dedicate greatest part of their time foraging and on exploratory behaviors such as grazing and rooting in their natural conditions. Furthermore, it was expected that pen environment and sex would influence time spent actively standing because of some pigs that were observed to be on heat, as these pigs would roam around sniffing and mounting others.

More time spent lying down compared to other activities may be a result of unavailability of space for other pigs to manoeuvre freely. Bakare et al [7] reported that increased time spent lying down could come directly from decreased time spent eating. More time spent on walking in male pigs under barren environment could have emanated when male pigs were walking around sniffing the floor. De Leeuw et al [11] referred this action as a substrate directed behavior. Substrate directed behavior may occur when pigs in a barren environment perceive that their pen environment is different from the other environment, thus, they spent time walking around looking for rootable objects.

The observation that female pigs in barren environment spent more time mounting could have been signs of boredom and females coming to heat because of sexual maturity. The observed similar time spent exploring substrates in enriched environment agrees with the commonly held view that when pigs are given access to substrates they spend considerate amount of time exploring them. The observation that male and female pigs in barren environment spent more time exploring pen fixtures could reflect rooting behavior in pigs. The finding that enriched pigs spent less time exploring pen fixtures could be due to that exploring pen fixtures was less satisfying as they were provided with rootable objects [12]. The observed interaction of pen environment and sex on skin lesions could be due to difficulty in avoiding an attack by an aggressor at a confined environment [13]. The finding that female pigs in barren environment had more lesions on the head compared to enriched females was expected. This could be because the provision of enrichment materials was expected to reduce the incidence of fighting since most of the pigs will be devoting their time exploring the substrates.

The finding that enriched females had less injuries on the neck and shoulder region than females in barren environment was expected because when pigs are fighting they position themselves so that their heads align on each other’s shoulders. Slamming of their heads into each other’s shoulders could have caused more cuts or injuries on the neck and shoulder region of females in barren environment [7]. More, so whilst fighting pigs attempt to target the head, neck and shoulders and ears of their opponents using bites and slashes [8]. Possibly this could have resulted in accumulation of high lesions in neck and shoulder region in females in barren environment. A redirected behavior of exploring the objects introduced in the pen may strongly explain the high probability of reduced skin lesion on other parts of the body in enriched female pigs [14].

## CONCLUSION

The study provides insights into management of Windsnyer pigs under confinement to improve pig production. The results showed a close agreement with other studies that providing objects could be an effective strategy to increase explorative behavior. Regarding skin injuries, pen enrichment reduced the number of injuries, especially for female Windsnyer pigs. Pen enrichment led to reduced time spent on aggressive interactions. Female pigs in enriched pens spent less time bullying each other than male pigs. These findings suggest that enriching the environment improves behavior of indigenous pig by reducing the rate at which anomalous behavior occur.

## Figures and Tables

**Table 1:** Ethogram of behavioral activities in barren and enriched environments

Behaviors	Description of behaviours
Fighting	Mutual pushing or lifting pen mate and pen mate retaliates
Bullying	If one pig is dominant over the other in a fight
Exploring substrates	Sniffing, rooting or pushing the ball or chewing the ball
Exploring pen fixtures	Rooting, sniffing, touching the walls or the ground of the pen except object or substrate
Standing active	Body weight supported by all four legs
Lying down	Body weight supported by belly side
Walking	Moving from one position to another position within a pen
Eating	Consumption of feed material from the feeder
Drinking	Drinking from the nipple drinker
Mounting	Placing front hoofs on the back of the pen mate
Head rubbing	When pigs rub heads without causing harm to each other
Others	When the focal pig is not involved in any of the listed behaviours

**Table 2:** Interaction between pen environment and sex on physiological responses of Windsnyer pigs

Variables	Pen environment	Sex		Covariate	p-value		
		
		Male	Female	*β1*	P	S	P×S
HR (beats/min)	Enriched	82.1±0.69^a^	82.5±0.73^a^	**	***	ns	ns
	Barren	88.4±0.66^b^	83.4±0.73^b^				
RR (beats/min)	Enriched	18.7±0.32^a^	18.5±0.34^a^	*	*	ns	*
	Barren	18.8±0.31^a^	19.9±0.34^b^				
RT (°C)	Enriched	38.4±0.06^a^	38.7±0.06^b^	*	ns	ns	***
	Barren	38.7±0.06^b^	38.5±0.06^a^				

HR, heart rate (beats/min); RR, respiration rate (beats/min); RT, rectal temperature 480 (°C); P, pen environment; S, sex; P×S, pen environment and sex interaction; not significant.

Level of significance:

*** p<0.001;

* p<0.05; ns, p>0.05.

ab Values with different superscripts across the treatments differ significantly (p<0.05).

**Table 3:** Percentage of observation time spent performing different behaviors by pigs in barren and enriched environments

Behaviour	Environment				SEM	p-value		

	Enriched		Barren					
		
	Male	Female	Male	Female		P	S	P×S
Feeding	13.1	13.3	13.5	13.1	1.62	ns	ns	ns
Drinking	4.6	4.8	4.5	4.8	0.99	ns	ns	ns
Fighting	3.1^a^	2.9^a^	5.8^b^	5^b^	1.49	***	ns	ns
Bullying	3.1^b^	2.0^a^	4.4^b^^c^	3.8^b^	1.51	***	ns	*
Lying down	26.0^ab^	27.9^b^	27.7^b^	24^a^	5.27	ns	ns	*
Standing active	12.1	13.5	14.2	13.3	2.93	ns	ns	ns
Walking	12.1^a^	13.5^b^	14.2^b^	13.3^ab^	2.43	*	ns	*
Mounting	3.3^ab^	2.7^a^	3.9^b^	3.5^b^	1.21	ns	ns	*
Head thrusting	3.1	3.1	2.7	2.9	1.31	ns	ns	ns
Explore substrates	10.6^a^	12.1^a^	-	-	2.99	ns	ns	-
Explore pen fixtures	7.3^a^	7.1^a^	12.9^b^	12.9^b^	2.63	***	ns	ns
Others	4.0^a^	4.4^a^	5.8^b^	5^a^	2.57	ns	ns	*

P, pen environment; S, sex; P×S, pen environment and sex interaction; not significant.

Level of significance

*** p<0.001;

* p<0.05;

ns, p>0.05.

ab Values with different superscripts across the treatments differ significantly (p<0.05).

**Table 4:** Interaction between pen environment and sex on skin lesions (mean±standard error)

Sex	Head region		Neck and shoulder region		Other parts	
		
	Enriched	Barren	Enriched	Barren	Enriched	Barren
Male	0.23±0.03^a^	0.22±0.03^a^	0.34±0.03^b^	0.42±0.03^b^	0.37±0.02^b^	0.37±0.02^b^
Female	0.33±0.03^a^	0.41±0.03^b^	0.21±0.03^a^	0.39±0.03^b^	0.23±0.02^a^	0.32±0.02^b^

ab Values with different superscripts across the treatments differ significantly (p<0.05).
